# Supplementary material for: Role and mechanism of NCAPD3 in promoting malignant behaviors in gastric cancer
Source: Front Pharmacol. 2024 Apr 22;15:1341039. doi: 10.3389/fphar.2024.1341039 (PMC11070777; doi:10.3389/fphar.2024.1341039)
Supplement: Supplementary file 11 [file DataSheet2.ZIP › GSEA/Transcipt factor/my_analysis.Gsea.1599461814734/AR_Q6.html]

Details for gene set AR\_Q6[GSEA]

|  || Dataset | filtered\_dataset.sample\_info.cls#WT\_versus\_NCAPD3\_MUT |
| Phenotype | sample\_info.cls#WT\_versus\_NCAPD3\_MUT |
| Upregulated in class | WT |
| GeneSet | AR\_Q6 |
| Enrichment Score (ES) | 0.49251947 |
| Normalized Enrichment Score (NES) | 1.9878088 |
| Nominal p-value | 0.0072859745 |
| FDR q-value | 0.21175388 |
| FWER p-Value | 0.663 |
Table: GSEA Results Summary

  

Fig 1: Enrichment plot: AR\_Q6      
 Profile of the Running ES Score & Positions of GeneSet Members on the Rank Ordered List

  

| SYMBOL | TITLE | RANK IN GENE LIST | RANK METRIC SCORE | RUNNING ES | CORE ENRICHMENT || 1 | 51585 | PCF11 | 3 | 1.212 | 0.1064 | Yes |
| 2 | 2243 | FGA | 18 | 1.039 | 0.1893 | Yes |
| 3 | 7170 | TPM3 | 24 | 1.018 | 0.2769 | Yes |
| 4 | 7464 | CORO2A | 138 | 0.757 | 0.2636 | Yes |
| 5 | 10929 | SRSF8 | 150 | 0.735 | 0.3216 | Yes |
| 6 | 54516 | MTRF1L | 187 | 0.687 | 0.3573 | Yes |
| 7 | 23001 | WDFY3 | 240 | 0.642 | 0.3774 | Yes |
| 8 | 1161 | ERCC8 | 255 | 0.625 | 0.4233 | Yes |
| 9 | 4281 | MID1 | 294 | 0.595 | 0.4493 | Yes |
| 10 | 8573 | CASK | 368 | 0.554 | 0.4465 | Yes |
| 11 | 5930 | RBBP6 | 390 | 0.543 | 0.4800 | Yes |
| 12 | 146691 | TOM1L2 | 472 | 0.490 | 0.4657 | Yes |
| 13 | 140688 | NOL4L | 495 | 0.475 | 0.4925 | Yes |
| 14 | 285636 | C5orf51 | 692 | 0.382 | 0.3861 | No |
| 15 | 10492 | SYNCRIP | 779 | 0.334 | 0.3543 | No |
| 16 | 6732 | SRPK1 | 835 | 0.291 | 0.3408 | No |
| 17 | 667 | DST | 886 | -0.303 | 0.3320 | No |
| 18 | 10098 | TSPAN5 | 1112 | -0.485 | 0.2139 | No |
Table: GSEA details [plain text format]

  

Fig 2: AR\_Q6      
 Blue-Pink O' Gram in the Space of the Analyzed GeneSet

  

Fig 3: AR\_Q6: Random ES distribution      
 Gene set null distribution of ES for **AR\_Q6**
